# The Emerging Role of Deubiquitinases in Cell Death

**DOI:** 10.3390/biom12121825

**Published:** 2022-12-06

**Authors:** Zhuan Zhou, Xinxin Song, Rui Kang, Daolin Tang

**Affiliations:** Department of Surgery, UT Southwestern Medical Center, Dallas, TX 75390, USA

**Keywords:** cell death, deubiquitinases, E3 ligases, protein degradation, post-translational modification, ubiquitin

## Abstract

Regulated cell death (RCD) is a signal-controlled process that not only eliminates infected, damaged, or aged cells but is also implicated in a variety of pathological conditions. The process of RCD is regulated by intracellular proteins that undergo varying levels of post-translational modifications, including mono- or polyubiquitination. Functionally, ubiquitination can affect protein abundance, localization, and activity. Like other post-translational modifications, ubiquitination is a dynamic and reversible process mediated by deubiquitinases, a large class of proteases that cleave ubiquitin from proteins and other substrates. The balance between ubiquitination and deubiquitination machinery determines cell fate under stressful conditions. Here, we review the latest advances in our understanding of the role of deubiquitinases in regulating the main types of RCD, including apoptosis, necroptosis, pyroptosis, and ferroptosis. This knowledge may contribute to identifying new protein degradation-related prognostic markers and therapeutic targets for human disease.

## 1. Introduction

Cell death is not only a physiological process that maintains tissue development and body homeostasis but also a pathological mechanism that disrupts normal organ function and stimulates inflammatory responses. Cells may die by accidental cell death (ACD) or regulated cell death (RCD) [1]. Unlike ACD, which is an uncontrolled process, RCD is fine-tuned through multiple signaling pathways and molecular mechanisms. Since the discovery of apoptosis by pathologists, many other forms of non-apoptotic RCDs (e.g., necroptosis, pyroptosis, and ferroptosis) have recently been identified through drug screening and now play a complex role in various pathological conditions [2]. Targeting cell death pathways is a strategy for the treatment of human diseases. One pharmacological approach is the use of small-molecule compounds to enhance or inhibit post-translational modifications (PTMs) of proteins involved in cell death machinery. PTMs are enzymatically mediated modifications of amino acid side chains after protein biosynthesis that increase protein functional diversity. Ubiquitination is a well-studied reversible form of PTM in which ubiquitin proteins are linked to substrate proteins through enzymatic reactions. In contrast, a family of deubiquitinases (DUBs) can remove ubiquitin from substrate proteins. The interaction between ubiquitination and deubiquitination is an important mechanism for regulating protein abundance, localization, and activity, thereby affecting cell survival and death. In this review, we discuss the emerging roles of DUBs in major types of RCDs. In addition to introducing the basic process of ubiquitination and deubiquitination, we will focus on summarizing the protein targets and signaling pathways of cell death controlled by DUBs.

## 2. The Ubiquitination-Deubiquitination Cycle

Ubiquitination is a covalent PTM of proteins with the protein ubiquitin (UB), which contains 76 amino acids. This process is mediated by a cascade of ubiquitination-activating E1, ubiquitin-conjugating enzymes E2 (E2s), and ubiquitin ligase E3 enzymes (E3s) [3]. In humans, approximately 30 E2s and 600 E3s mediate ubiquitin attachment to selected target proteins, ensuring the specificity of substrate selection [4]. The most common form is the formation of an isopeptide bond between the C-terminus of ubiquitin and a lysine side chain in the target protein. Less common forms are the C-terminal glycine attached to the N-terminal or serine or threonine side chain of the protein [5]. Moreover, ubiquitin itself can be modified at its N-terminus methionine (Met1) or one of its seven internal lysine residues of the ubiquitin (Lys6, Lys11, Lys27, Lys29, Lys33, Lys48, or Lys63) through isopeptide bond formation with carboxy-terminal glycine, allowing the assembly of diversified polyubiquitin chains [6]. The polyubiquitin chain may contain mixed linkages and two or more branches, as well as linkages between ubiquitin and ubiquitin-like proteins (UBLs), including small ubiquitin-like modifier (SUMO) and neuronal precursor cell-expressed developmentally downregulated protein 8 (NEDD8) [7]. Different ubiquitin chains or UBLs modifications formulate a “UB code” read by the cognate binding domains, which controls the fates of modified proteins by regulating protein stability, interactions, and localization [6].

**Figure 1:**
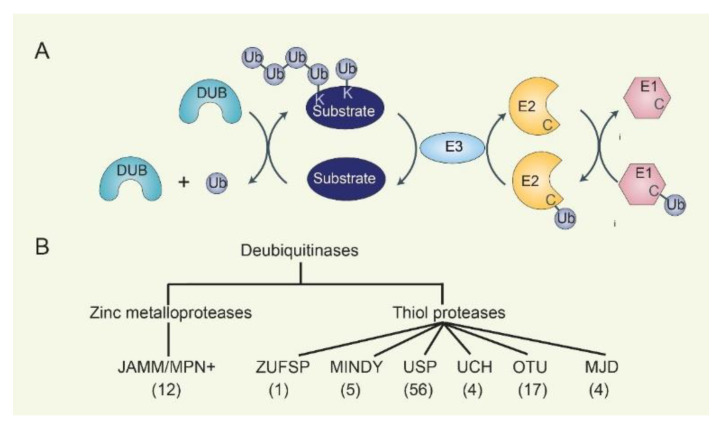
**The cascades of ubiquitination/deubiquitination and families of DUBs**. (**A**) E1, E2, and E3 cascades add mono- or polyubiquitin chains to substrates. DUB removes substrate-bound ubiquitin. (**B**) DUB contains seven subgroups: JAMM/MPN+, ZUSP, MINDY, USP, UCH, OTU, and MJD, depending on the characteristics of the conserved domains. The number of genes in each family is indicated.

The reversal of ubiquitin conjugation of targeted proteins relies on DUBs, which catalytically cleave single ubiquitin or polyubiquitin chains from proteins, edit ubiquitin chains, and process ubiquitin precursors (Figure 1). Specifically, DUBs can directly remove ubiquitin chains from ubiquitinated proteins to prevent their degradation by the proteasome or ubiquitin signaling. DUBs can also inhibit the ubiquitination process by interfering with the E2-Ub intermediate or by counteracting the activity of E3s. In addition, some DUBs can trim and edit ubiquitin chains to maintain cellular ubiquitin pool homeostasis. The human DUBs have about 100 members, which can be classified into 7 families according to evolutionary conservation: ubiquitin-specific proteases (USPs), ubiquitin COOH-terminal hydrolases (UCHs), ovarian tumor proteases (OTUs), Machado–Josephin domain-containing proteases (MJDs), the JAB1/MPN/MOV34 family (JAMMs, also known as MPN+ and hereafter referred to as JAMM/MPN+), motif interacting with Ub-containing novel DUB family (MINDYs), and ZUFSP family [8,9]. USP, UCH, OTU, MJD, MINDY, and ZUFSP families are thiol proteases, while the JAMM/MPN+ family comprises zinc metalloproteases [10]. Overall, understanding the cellular and tissue expression specificity of DUB and E3 family expression is key to elucidating the context-dependent role of the ubiquitin–proteasome system involved in substrate degradation.

## 3. DUBs in Apoptosis

**Figure 2:**
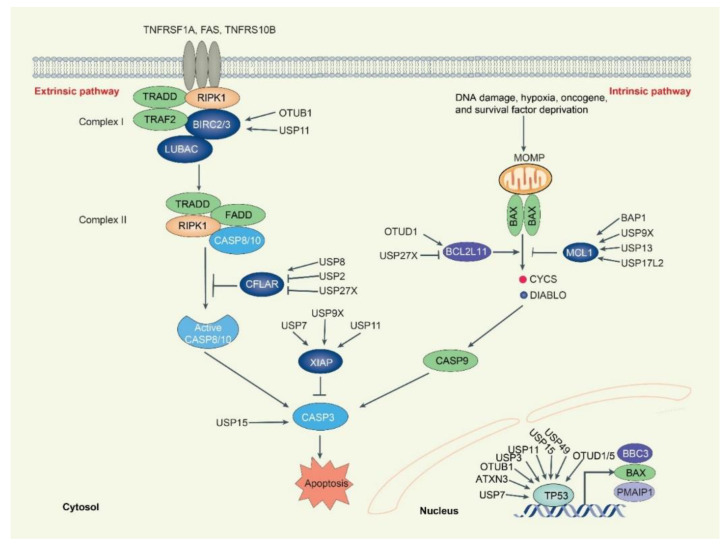
**Overview of DUBs-mediated regulation of apoptosis**. Activation of the extrinsic apoptotic pathway forms complex 1 (TRADD-TRAF2-RIPK1-BIRC2/3 complex). BIRC2/3 regulates RIPK1 and its autoubiquitination, whereas USP11 and OTUB1 deubiquitinate and stabilize BIRC2/3. Active RIPK1, TRADD, FADD and CASP8/10 form complex II to trigger the CASP8/10-CASP3 cascade of the apoptotic process. The CASP8/10 inhibitor CFLAR is rapidly renewed and deubiquitinated by DUB USP8. USP2 and USP27X reduce CFLAR expression by deubiquitinating their E3 ligases TRIM28 and ITCH. The intrinsic apoptotic pathway is controlled at the level of the BCL2 family. The pro-apoptotic protein BCL2L11 and the anti-apoptotic protein MCL1 proteins are tightly controlled by DUBs. While OTUD1 stabilizes BCL2L11, USP27x promotes the binding of BCL2L11 to E3 ligases to facilitate its degradation. Three DUBs (USP9X, USP13, and USP17L2) deubiquitinate MCL1, while BAP1 promotes MCL1 transcription through H2A deubiquitinate. The extrinsic/intrinsic apoptotic signal is incorporated into CASP3, which is deubiquitinated by USP15. The TP53 protein is responsible for the expression of BBR3, BAX, and PMAIP1, which cause apoptosis. The expression of TP53 is controlled by as many as nine DUBs, including USP3/7/11/15/22/49, OTUB1, OTUD1/5, and ATXN3.

Apoptosis is an evolutionarily conserved form of RCD that typically involves caspases, a family of cysteine-aspartic proteases. Morphologically, apoptotic cells exhibit cell shrinkage, membrane integrity, membrane blebbing, chromatin condensation, and cell fragmentation to form apoptotic bodies [11]. Mechanistically, apoptosis can be divided into extrinsic and intrinsic pathways with different initiation signals. The extrinsic pathway is triggered by death ligands (e.g., FASLG, TNF, and TNFSF10), which bind to their death receptors (e.g., FAS, TNFRSF1A [TNFR1], and TNFRSF10B [DR5]), leading to the recruitment of the adaptor protein FAS-associated via death domain (FADD) and tumor necrosis factor receptor type 1-associated DEATH domain protein (TRADD) to CASP8 or CASP10 [12]. After their recruitment, CASP8 or CASP10 is activated and initiates apoptosis by cleaving downstream effector caspases (including CASP3, CASP6, and CASP7), eventually resulting in apoptosis (Figure 2). The intrinsic pathway, also known as mitochondrial apoptosis, is triggered by various stress signals, such as oncogene expression, genome damage, hypoxia, and nutrient deprivation. These stimuli lead to mitochondrial outer membrane permeabilization (MOMP), which opens the mitochondrial permeability transition pore (MPTP) and subsequent release of mitochondrial proteins, including cytochrome C (CYCS), diablo IAP-binding mitochondrial protein (DIABLO; also known as SMAC), apoptosis inducing factor mitochondria associated 1 (AIFM1; also known as AIF), and endonuclease G (ENDOG), into the cytoplasm or nucleus. Cytoplasmic CYCS and DIABLO activate CASP9 to induce apoptotic cell death, whereas nuclear AIFM1 and ENDOG induce nuclear DNA fragmentation to trigger apoptosis in a caspase-independent manner [2].

CASP8 and FADD-like apoptosis regulator (CFLAR, also known as c-FLIP), a competitive mimetic of pro-CASP8, is a major anti-apoptotic protein that suppresses cytokine- and chemotherapy-induced apoptosis [13]. CFLAR binds to FADD and/or CASP8 or CASP10 to prevent death-inducing signaling complex (DISC) formation and subsequent activation of the caspase cascade. Human CFLAR has three protein isoforms, CFLAR_L_, CFLAR_S_, and the rarely detected CFLAR_R_. The E3 ubiquitin ligase itchy E3 ubiquitin-protein ligase (ITCH) couples mitogen-activated protein kinase 8 (MAPK8, also known as JNK) activation to TNF-induced cell death by inducing CFLAR_L_ turnover via ubiquitination and proteasomal degradation [14]. The ubiquitin E3 ligase DELTEX1 (DTX1) enhances CFLAR degradation and FASLG- or TNFSF10-induced apoptosis in gastric cancer cells [15]. USP8 prevents extrinsic apoptosis, initiated by the ligation of anti-FAS antibody, TNF, or TNFSF10 to their specific receptors, through the direct deubiquitylation and stabilization of CFLAR_L,_ rather than regulating the expression or surface availability of death receptors in cervical cancer and melanoma cells [16]. The overexpression of DUB ubiquitin-specific protease 27 X-Linked (USP27X) leads to the loss of the CFLAR_L_ protein and sensitizes extrinsic apoptosis in melanoma cells. USP27X interacts with the E3-ligase tripartite motif containing 28 (TRIM28) and reduces the ubiquitination of E3-ligases TRIM28, but not ITCH and DTX1, which leads to decreased CFLAR protein [17]. The DUB ubiquitin-specific protease 2 (USP2) also promotes MAPK8-mediated and TNF-induced activation of ITCH and subsequent CFLAR_L/S_ degradation. The knockdown of USP2 protects hepatocytes from TNF-induced apoptosis by interference with CFLAR signaling [18]. These findings establish that USP8, USP27X, and USP2 play major roles in regulating CFLAR degradation during apoptosis.

Cellular inhibitors of apoptosis (c-IAP) proteins are E3 ubiquitin ligases that promote the assembly of polyubiquitin chains on themselves and are critical regulators of TNFR1 signaling [19]. The c-IAP members baculoviral IAP repeat containing 2 and 3 (BIRC2 and 3, also known as c-IAP1 and c-IAP2) are recruited to the TNFR-associated factor 2 (TRAF2)-receptor-interacting serine/threonine kinase 1 (RIPK1) complex to mediate the ubiquitination of RIPK1 predominantly with K63 and K11 ubiquitin linkages [20]. The DUB OTU domain-containing ubiquitin aldehyde-binding protein 1 (OTUB1) is a BIRC2-associated deubiquitinating enzyme that regulates BIRC2 stability. OTUB1 disassembles K48-linked polyubiquitin chains on BIRC2 and inhibits TNF superfamily member 12 (TNFSF12)- and c-IAP antagonist-stimulated caspase activation and cell death [21]. Ubiquitin-specific protease 11 (USP11) is a DUB that directly stabilizes BIRC3 and protects BIRC3 from DIABLO mimetic-mediated degradation. USP11 downregulates sensitized TNFSF10 and SMAC mimetic BV6-induced apoptosis and inhibits tumor growth [22].

The X-linked inhibitor of apoptosis (XIAP) is an endogenous proteolytic activity inhibitor of CASP3, CASP7, and CASP9. DIABLO released from mitochondria activates CASP9 by inhibiting XIAP activity [23]. XIAP contains RING domain on the carboxyl-terminus, which leads it to undergo self-ubiquitylation and targets BCL2 degradation [24]. The E3 ligase siah E3 ubiquitin protein ligase 1 (SIAH1) also targets XIAP for ubiquitylation degradation. On the other hand, several DUBs, including USP11, ubiquitin-specific peptidase 9 X-linked (USP9X), and ubiquitin-specific protease 7 (USP7), stabilize XIAP [25,26,27]. USP11 binds to Leu207 on the BIR2 domain of XIAP to stabilize XIAP, thereby inhibiting anoikis and apoptosis and promoting mammary tumor initiation and progression [25]. USP9X, a mitotic DUB, can bind to the Gly188 on the BIR2 domain of XIAP to stabilize XIAP, thereby increasing resistance to mitotic spindle poisons in primary human aggressive B-cell lymphoma [26]. USP7 physically interacts and stabilizes XIAP by employing its DUB activity, which is associated with doxorubicin resistance to abrogate apoptosis in colorectal cancer and glioma cells [27]. Thus, XIAP deubiquitination is required to reset its activity, and attenuation of XIAP may contribute to tumor suppressor function by inducing apoptosis.

The intrinsic mitochondrial apoptosis pathway is primarily controlled by the BCL2 family. The BCL2 family can be split into pro-survival/anti-apoptotic (BCL2, BCL2L1, BCL2L2, BCL2A1, and MCL1), effector (BAK1, BAX, and BOK), BH3-only activator (BCL2L11/BIM, BID, BBC3/PUMA), and sensitizer (PMAIP1/NOXA), BAD, BMF, BIK, and HRK) proteins [28]. MCL1 is the most labile of the pro-survival protein that is rapidly degraded by the K48-linked polyubiquitin–proteasome pathway in the presence of therapeutic pressure. MCL1 is targeted and degraded by several ubiquitin ligases, including HECT, UBA, and WWE domain-containing E3 ubiquitin-protein ligase 1 (HUWE1), F-box and WD repeat domain-containing 7 (FBXW7), beta-transducin repeat containing E3 ubiquitin protein ligase (BTRC), membrane-associated ring-CH-type finger 5 (MARCHF5), and anaphase-promoting complex/cyclosome—cell division cycle 20 (APC/C^Cdc20^) [29]. In counter to E3 ligase, several DUBs, including USP9X, ubiquitin-specific peptidase 17 such as family member 2 (USP17L2), and USP13, enhance cell survival by deubiquitinating MCL1 and limiting its turnover in certain patient tumors [30,31,32]. USP9X can bind and remove polyubiquitin chains from MCL1 targeted for degradation, and its expression correlates with MCL1 overexpression in follicular lymphoma, diffuse large B-cell lymphoma, and multiple myeloma. Unlike USP9X, which exhibits tissue-specific expression primarily in the brain and immune system, USP13 regulates MCL1 turnover in lung and ovarian cancers [30,32]. However, USP13 is a relatively less effective DUB for removing the K40 sites K48-polyubiquitin chain. Alternatively, the more efficient DUB USP17L2 controls MCL1 turnover in ovarian cancer cells [31]. The DUB BRCA1-associated protein 1 (BAP1), a widely expressed DUB for histone H2A, promotes the expression of MCL1 and BCL2 [33]. BAP1 inactivation causes apoptosis in mouse embryonic stem cells, fibroblasts, liver, and pancreatic tissue, but not in melanocytes and mesothelial cells [33].

The BCL2 family protein BCL2L11 is a member of the pro-apoptotic group of BH3-only proteins. BCL2L11 is degraded in response to a major oncogenic pathway by E3 ligase APC/C^CDC20^ and BTRC [34,35]. Furthermore, USP27x is present in complex with BCL2L11 and BTRC, which facilitates BCL2L11 degradation in response to mitogen-activated protein kinase (MAPK)/extracellular signal-regulated kinase (ERK) signaling and sensitizes human cancer cells to chemotherapeutic drugs [36]. The DUB ovarian tumor domain-containing protein 1 (OTUD1), a DUB belonging to the OTU protein family, is upregulated by melatonin at the mRNA and protein levels, resulting in the deubiquitination at the lysine 3 residue of BCL2L11 and subsequent stabilization of BCL2L11. BCL2L11 expression levels correlate with OTUD1 levels in patients with renal clear cell carcinoma, highlighting OTUB1 as a potential biomarker for predicting drug response [37].

Other protein degradation targets in apoptosis are CASP3 and tumor protein p53 (TP53, also known as p53). The ubiquitination of CASP3 at its N-terminal domain is mediated by E3 ligase BTRC [38]. XIAP has E3 ligase activity and promotes proteasomal degradation of CASP3 [39]. In contrast, DUB USP15 counteracts the activity of the BTRC, thereby increasing the stability and activity of CASP3 during paclitaxel-induced apoptosis [40]. Transcription factor TP53 is activated in response to many stress stimuli and further induces BBC3, BAX, and PMAIP1 expression to induce apoptosis [41]. The expression of TP53 is controlled by the E3 ligase MDM2 [42]. USP10, a cytoplasmic DUB, directly deubiquitinates TP53 and regulates the subcellular localization and stability of TP53 by antagonizing the action of MDM2 [43]. Ataxin-3 (ATXN3), a member of the MJD DUB family, also directly binds to native and polyubiquitinated TP53 and deubiquitinates and stabilizes TP53 by repressing its degradation through the ubiquitin–proteasome pathway [44]. The DUB USP7 deubiquitinates MDM2 and leads to stabilization of TP53, which limits TP53-dependent expression of pro-apoptotic BCL2 family members [45]. OTUB1 also inhibits MDM2-mediated ubiquitination of TP53 in cancer cells, but its catalytic activity is not required for these effects. In contrast with USP7, OTUB1 may interfere with the ubiquitination of TP53 by inhibiting the MDM2 cognate E2 UBE2D1 [46]. USP3 and USP11 deubiquitinate and stabilize TP53, promoting normal cellular transformation or mediating DNA damage [47]. USP15 increases TP53 stability and subsequent TP53-mediated cyclin-dependent kinase inhibitor 1A (CDKN1A) gene expression to inhibit proliferation of human osteosarcoma cell line U2OS [48]. In contrast, USP15 controls the protein expression of TP53-R175H, but not TP53 WT, through the ubiquitin-mediated lysosomal pathway in ovarian cancer cells [49]. In addition, ubiquitin-specific protease 49 (USP49) binds and stabilizes TP53 through deubiquitination and participates in DNA damage response by forming a positive feedback loop with TP53 [50]. Other DUBs, OTUD1 and OTUD5, are also involved in the deubiquitination and stabilization of TP53 in response to DNA damage stress in U2OS cells [51,52]. These findings suggest a context-sensitive role for the DUB family in regulating TP53 stability and subsequent function in apoptosis.

## 4. DUBs in Necroptosis

**Figure 3:**
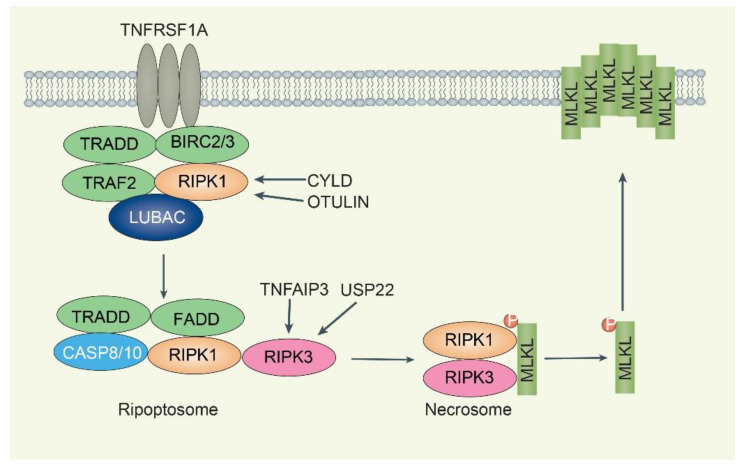
**Overview of DUBs-mediated regulation of necroptosis**. TNFRSF1A activates recruitment complex 1 (TRADD-TRAF2-RIPK1-BIRC2/3 complex), and RIPK1 is a core regulatory kinase regulated by ubiquitination. The DUBs CYLD and OTULIN stabilize RIPK1 for further ripoptosome formation (complex IIb). The ripoptosome complex has TRADD, FADD, CASP8/10, and RIPK1/3. If the activity of CASP8/10 is inhibited, RIPK1/3 and MLKL form necrosomes in which MLKL is phosphorylated by RIPK3. Translocation of active MLKL to the plasma membrane results in membrane rupture. USP22 and TNFAIP3 reduce RIPK3 K518 and K5 ubiquitination, respectively. TNFAIP3 inhibits necroptosis, whereas USP22 accelerates necroptosis.

Necroptosis is a caspase-independent regulated necrosis that is more pro-inflammatory than apoptosis and is characterized by necrotic morphological changes, including cellular/organelle swelling, rupturing of the plasma membrane, and moderate chromatin condensation [53]. A plethora of different stimuli, including members of the TNFR superfamily, pattern recognition receptors, T cell receptors, and multiple chemotherapeutic drugs, can activate the necroptotic death pathway [54]. The most defined pathway for inducing necroptosis is through the TNF/TNFR1-complex II signaling pathway. The binding of TNF to TNFR1 induces a conformational change in TNFR1 trimers, leading to the recruitment of multiple proteins to form complex I, including receptor-interacting protein kinase 1 (RIPK1), TRADD, BIRC2, BIRC3, TRAF2, by TNFR1 (Figure 3) [54]. RIPK1 in complex I is polyubiquitinated by BIRC2 or BIRC3 and subsequently induces the canonical nuclear factor kappa B (NF-κB) pathway. If RIPK1 is deubiquitinated by the DUB cylindromatosis (CYLD), which limits sustained activation of NF-κB signaling and leads to a tendency to form complex II [55]. Complex II is a cytoplasmic death-inducing signaling complex comprising RIPK1, TRADD, CASP8, and FADD, which is also referred to as “ripoptosome” [56]. In ripoptosome, active CASP8 cleaves both RIPK1 and RIPK3, resulting in their inactivation, and the pro-apoptotic caspase activation cascade is initiated, ultimately leading to apoptosis execution. The CASP8-CFLARs heterodimers do not have apoptosis-inducing activity, whereas blocking CASP8 activity by viral protein CrmA or pharmacological agents Z-VAD-FMK or Z-IETD-FMK or by the depletion of CASP8 promotes necroptosis [57]. The combination of TNF, the pan-caspase inhibitor Z-VAD-FMK (inhibiting CASP8 activity), and a SMAC mimetic (BIRC2/3 inhibitor) can induce necroptosis since stimulation of TNFR1 by TNF alone does not form complex II [20]. The necrosome is a protein complex that contains core components RIPK1, RIPK3, and mixed-lineage kinase domain-like pseudokinase (MLKL) [58]. The RIPK3-mediated phosphorylation of MLKL results in its oligomerization and subsequent translocation into the plasma membrane, causing membrane rupture. Necrosome formation and/or activation can be blocked by RIPK1 inhibitor necrostatin-1 (Nec-1), MLKL inhibitor necrosulfonamide (NSA), and multiple RIPK3 inhibitors [58].

In complex I, RIPK1 is extensively modified by different types of ubiquitin chains, including M1, K11, and K63, mediated by E3 ubiquitin ligases, such as BIRC2/3 and linear ubiquitin chain assembly complex (LUBAC) [59]. The necroptotic RIPK1 K115 ubiquitination is important for maintaining RIPK1 kinase activity in the necrosome complex [60]. To date, M1-/K63-linked ubiquitin chains seem to be predominantly conjugated to RIPK1 during TNF-induced necroptosis [60]. The LUBAC, which consists of ring finger protein 31 (RNF31, also known as HOIP), SHANK-associated RH domain interactor (SHARPIN), and RANBP2-type and C3HC4-type zinc finger containing 1 (RBCK1, also known as HOIL-1L), mediates M1-linked ubiquitination of RIPK1 within the necrosome, while the DUB OTU deubiquitinase with linear linkage specificity (OTULIN) removes M1-linked chains from RIPK1 during necroptosis [61]. DUB CYLD is recruited to complex I via the LUBAC component RNF31 to promote necroptosis by removing the K63/M1 ubiquitin chain of RIPK1 [62,63]. CYLD is also responsible for deubiquitinating TRAF2 during necroptosis, stopping constitutive TRAF2 associated with MLKL [64]. The pro-necroptotic effect of CYLD on RIPK1 K63/M1 ubiquitination is opposed by the TNFAIP3 interacting protein 1 (TNIP1)/TNF alpha-induced protein 3 (TNFAIP3, also known as A20) complex, which interacts with M1 chains and prevents their removal [65].

RIPK3 ubiquitination is involved in the regulation of necroptosis. The K5, K42, K55, K197, K302, K351, K364, K363, K469, K501, and K518 sites are critical ubiquitination modification sites [66,67,68,69,70,71]. The E3 ligase STIP1 homology and U-box containing protein 1 (STUB1, also known as carboxy terminus of HSP70-interacting protein/CHIP) can regulate cell necroptosis through ubiquitylation- and lysosome-dependent RIPK3 degradation by K55 and K363 ubiquitination [66]. The pellino E3 ubiquitin-protein ligase 1 (PELI1) mediates K48-linked polyubiquitylation of RIPK3 on lysine 363, leading to proteasomal degradation of RIPK3 [67]. The E3 ligase tripartite motif containing 25 (TRIM25) binds to RIPK3, promotes polyubiquitination of K48-linked RIPK3 at residue K501, and negatively regulates RIPK3 stability through the ubiquitin–proteasome degradation pathway [68]. The E3 ligase Parkin (PRKN) is implicated in RIPK3 ubiquitination at K197, K302, and K364 residues conjugated K33-linked ubiquitin chains during TNF-induced necroptosis [69]. On the other hand, the DUB TNFAIP3 decreases RIPK3 ubiquitination and reduces the RIPK1:RIPK3 interaction, thereby suppressing TNF-induced necroptosis [70]. Mass spectrometry identifies K5 of RIPK3 as a ubiquitination site, specifically in TNFAIP3-knockout mouse cells undergoing necroptosis [70]. The DUB ubiquitin-specific protease 22 (USP22) controls RIPK3 phosphorylation and ubiquitination of K42, K351, and K518. Moreover, K518 ubiquitination of RIPK3 reduces its interaction with necrosomes and inhibits TNF/SMAC mimetic/Z-VAD-FMK-induced necroptosis, which is reversed by USP22 [71].

Taken together, these independent studies highlight that DUBs can shape necroptosis sensitivity by modulating ubiquitin levels of RIPK1 or RIPK3, although whether MLKL protein stability is regulated by DUB remains poorly understood.

## 5. DUBs in Ferroptosis

**Figure 4:**
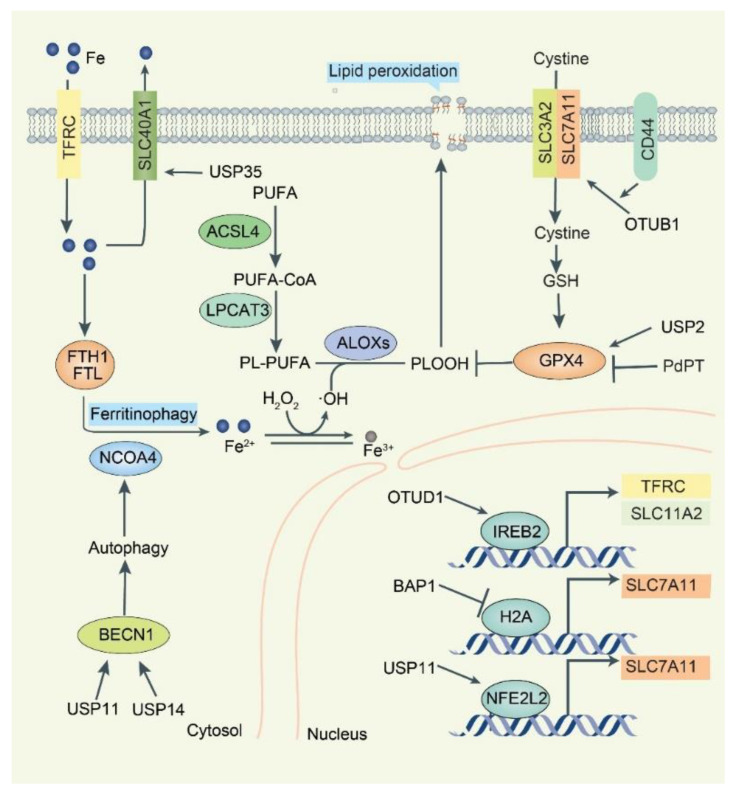
**Overview of DUBs-mediated regulation of ferroptosis**. Induction of iron-mediated ferroptosis depends on disruption of the balance of oxidants and antioxidants. SLC40A1 is the only cellular iron exporter. USP35 binds to SLC40A1 to stabilize its expression and inhibit ferroptosis. TFRC uptakes extracellular iron and stores iron through FTH1/FTL. Cell-destabilizing iron release from ferritin is dependent on NCOA4-dependent ferrotinophagy. Labile iron promotes the production of PL-OOH, which leads to membrane lipid oxidation and cell death. BECN1 promotes autophagy and ferrotinophagy. Two DUBs, USP11 and USP14, are responsible for BECN1 deubiquitination. SLC7A11 and SLC3A2 form the amino acid transport system xc–, which uptakes cystine into the cytosol, where it is rapidly converted to cysteine and used for glutathione synthesis. The stem cell marker CD44 promotes OTUB1-SLC7A11 association and stabilizes SLC7A11. Selenium enzyme GPX4 reduces PLOOH and inhibits ferroptosis via glutathione. DUB USP2 stabilizes GPX4, and the pan-DUBs inhibitor PdPT promotes GPX4 degradation. The transcription factor IREB2 is responsible for TFRC and SLC11A2 gene transcription. DUB OTUD1 binds and stabilizes IREB2, while BAP1 promotes SLC7A11 transcription through histone H2A deubiquitination. NFE2L2 is responsible for SLC7A11 transcription and USP11 stabilizes NFE2L2 protein.

Ferroptosis is a non-apoptotic RCD driven by oxidative stress-mediated lipid peroxidation [72]. It is morphologically characterized by mitochondrial abnormalities, including misshapen small mitochondria, reduced cristae, and condensed or ruptured outer membranes. The lethal accumulation of lipid peroxides from the peroxidation of polyunsaturated fatty acid phospholipids (PUFA-PLs) in cell membranes under conditions rich in iron and reactive oxygen species (ROS) is a major biochemical feature of ferroptosis (Figure 4) [73]. Multiple antioxidant systems, especially the SLC7A11-glutathione peroxidase 4 (GPX4) pathway, limit lipid peroxidation [74]. Accordingly, the process of ferroptosis can be inhibited by lipophilic radical traps, such as vitamin E, ferrostatin-1, and liproxstatin-1 [75]. In contrast, pharmacological inhibition of the SLC7A11-GPX4 pathway by small molecular compounds, such as erastin or RSL3, is now the classical approach to induce ferroptosis, although several pathological conditions, such as ischemia-reperfusion injury, hyperthermia, and pancreatitis, can activate ferroptosis [76,77]. Targeting ferroptosis is a new strategy for the treatment of human diseases, especially cancer [78].

Intracellular iron homeostasis relies on transferrin (TF)/transferrin receptor (TFRC) system-mediated iron uptake and solute carrier family 40 member 1 (SLC40A1, also known as ferroportin/FPN1)-mediated iron export [79]. DUB ubiquitin-specific protease 35 (USP35) interacts with SLC40A1 and maintains its protein stability to prevent iron overload and ferroptosis in lung cancer cells [80]. Of note, USP35 overexpression fails to affect tumorigenesis and ferroptosis under basal conditions, but reduces erastin/RSL3-triggered iron disturbance and ferroptosis, thereby facilitating lung cancer cell growth and tumor progression. The iron sensor, iron-responsive element-binding protein 2 (IREB2), directly binds to the RNA stem-loop structures in the 3′-untranslated region of mRNA and stabilizes transcripts of TFRC or solute carrier family 11 member 2 (SLC11A2), thereby increasing intracellular iron concentration [81]. The ubiquitin E3 ligase F-box/LRR-repeat protein 5 (FBXL5) promotes the ubiquitination and consequent degradation of IREB2, thereby limiting iron uptake and utilization under iron overload [82]. OTUD1 acts as a DUB for IREB2 and prevents its degradation, thereby promoting TFRC expression and increasing cellular iron uptake [83]. The activation of the OTUD1-IREB2-TFRC pathway increases intracellular iron concentration and enhances cellular susceptibility to ferroptosis and enhances host antitumor immunity in colorectal cancer [83]. Collectively, DUBs control the protein levels of various regulators of iron metabolism to influence ferroptosis.

Solute carrier family 7 membrane 11 (SLC7A11, also called xCT) is a key component of the amino acid transporter system xc^–^ that mediates cystine uptake and subsequent glutathione synthesis. GPX4 utilizes reduced glutathione (GSH) to reduce lipid hydroperoxides to lipid alcohols, thereby protecting cells from membrane lipid peroxidation and inhibiting ferroptosis [84]. Upon stimulation by ferroptosis inducers, gene transcription of SLC7A11 is activated mainly by NFE2-like BZIP transcription factor 2 (NFE2L2, also known as NRF2) and activating transcription factor 4 (ATF4) [85]. The tumor suppressor BRCA1-associated protein 1 (BAP1) encodes a nuclear deubiquitinating enzyme that interacts with several transcriptional factors and chromatin-modifying enzymes and plays a role in the epigenetic regulation of gene transcription [86]. BAP1 and its associated proteins form the polycomb repressive DUB complex, which mainly functions to remove monoubiquitin from ubiquitinated histone 2A at lysine 119 (H2Aub) on chromatin. BAP1 reduces H2Aub occupancy on the SLC7A11 promoter and represses SLC7A11 expression in a deubiquitin-dependent, but not NFE2L2- and ATF4-dependent manner [87]. Consequently, BAP1-mediated downregulation of SLC7A11 leads to elevated lipid peroxidation and ferroptosis [88]. In contrast, the DUB USP22, which antagonizes TP53 transcriptional activation by deubiquitinating sirtuin 1 (SIRT1), promotes the expression of SLC7A11 and inhibits ferroptosis-mediated cardiomyocyte death in a myocardial ischemia-reperfusion injury model [89]. DUB OTUB1 can also directly interact with SLC7A11 and stabilize SLC7A11 in a TP53-independent manner. The depletion of endogenous OTUB1 reduces SLC7A11 expression and promotes ferroptosis in human cancer cells, which results in growth inhibition of human bladder cancer cell T24 mouse tumor xenografts. Stem cell marker CD44 expression suppresses ferroptosis in cancer cells in an OTUB1-dependent manner by promoting the interaction between SLC7A11 and OTUB1 [90].

GPX4 is a selenoprotein that catalyzes lethal lipid hydroperoxides to nontoxic lipid alcohols in the presence of GSH as an essential cofactor. The E3 tripartite motif containing 46 (TRIM46) is one of the E3 ligases of GPX4 in human retinal capillary endothelial cells with high glucose treatment [91]. LUBAC mediates M1-linked ubiquitination of GPX4 to stabilize GPX4 under normal conditions or oxidative stress [92]. RSL3 induces rapid K48- and K63-linked ubiquitination of GPX4, which may provide the basis for the recruitment of LUBAC, followed by M1-linked ubiquitination of GPX4 to antagonize ferroptosis at early time points. DUB USP2 can remove K48- and K63-linked ubiquitin chains but not M1-linked ubiquitin chains from GPX4 [92]. Palladium pyrithione complex (PdPT), a broad-spectrum DUB (including USP7, USP10, USP14, USP15, USP25, and ubiquitin C-terminal hydrolase L5 (UCHL5) inhibitor, can also cause GPX4 protein ubiquitinated degradation in non-small cell lung cancer cells [93]. Therefore, the inhibition of DUB is a way to cause the degradation of GPX4 to trigger ferroptosis.

Transcription factor NFE2L2 plays a broad role in regulating the expression of anti-ferroptosis proteins, such as SLC7A11, GPX4, and metallothionein-1G (MT1G) [94,95]. E3 ligases, including KEAP1-CUL3-RBX1 complex, BTRC-SKP1-CUL1-RBX1 complex, and synoviolin 1 (SYVN1, also known as HRD) control NFE2L2 stability and accumulation. DUB USP11 reverses NFE2L2 polyubiquitination and stabilizes NFE2L2 to prevent oxidative stress-induced ferroptosis and promote tumorigenesis in non-small cell lung cancer cells [96]. However, it is unclear whether USP11 plays a similar role in regulating non-ferroptotic cell death through NFE2L2.

Autophagy is another degradation pathway with many intersections with the UPS pathway [97]. Although autophagy is generally a programmed cell survival pathway, certain selective autophagy can promote cell death, including ferroptosis [98,99,100]. For example, autophagy-mediated ferritin degradation (namely ferritinophagy) promotes ferroptosis by increasing the accumulation of intracellular iron [101]. BECN1 (also known as Atg6 in yeast) is a key regulator of autophagy that promotes ferroptosis by blocking SLC7A11 activity via forming the BECN1-SLC7A11 complex [102,103]. The DUB ubiquitin-specific protease 14 (USP14) could deubiquitinate BECN1 to enhance autophagy-dependent ferroptosis in A549 lung cancer cells [104]. Another DUB USP11 can trigger autophagy activation by stabilizing BECN1, promote ferritin degradation, and ultimately lead to iron-dependent ferroptosis in spinal cord ischemia-reperfusion injury [105]. Further understanding of the interaction between autophagy and UPS is expected to develop new strategies to control ferroptosis [106].

## 6. DUBs in Pyroptosis

Pyroptosis is a lytic form of RCD driven primarily by inflammatory caspases-mediated cleavage and activation of the gasdermin family (e.g., GSDMD) [107]. Its morphological features are necrotic-like changes with plasma membrane rupture and release of cellular contents that can activate inflammatory and immune responses [108]. Pyroptosis was originally described in infected immune cells (for example, macrophages and dendritic cells) and is now closely associated with the development of many inflammatory diseases, including sepsis and cancer [109,110,111,112]. The activation of pyroptosis is initiated by inflammasome assembly, including the canonical pathway of CASP1 and the non-canonical pathway activated by CASP11 or CASP4/5 in mice or humans, respectively [113,114]. Several apoptosis-related caspases, such as CASP3 and CASP8, also can promote pyroptosis in immune and cancer cells [115,116]. Canonical inflammasomes are usually formed by sensor proteins called pattern-recognition receptors (PRRs), an adaptor protein PYCARD (PYRIN and CARD domain containing, also called apoptosis-associated speck-like protein containing a CARD (ASC)), and an inactive pro-CASP1. There are five main types of inflammasomes in canonical pathways, namely NLRP3, AIM2, NLRP1, MEFV (also called PYRIN), or NLRC4 inflammasome [117]. The non-canonical inflammasome pathway is triggered by cytosolic LPS binding to CASP11. Inflammatory caspases can cleave the intact GSDMD into two parts, GSDMD-NT (the N-terminal domain of GSDMD) and GSDMD-CT (the C-terminal domain of GSDMD). GSDMD-NT acts as a mediator of pyroptosis, leading to pore formation, membrane lysis, and release of IL1 family and DAMPs (e.g., HMGB1) by translocating to the inner lobe of the plasma membrane [118,119,120]. This GSDMD-mediated membrane rupture process can be an active process, regulated by various proteins or signals, such as ninjurin 1 (NINJ1), lipid peroxidation, and Ca^2+^ influx [121]. Whether other gasdermin members (GSDMA, GSDMB, GSDMC, GSDME/DFNA5, and GSDMA3) require the same signaling pathway as GSDMD to mediate pyroptosis remains largely unknown [107].

**Figure 5:**
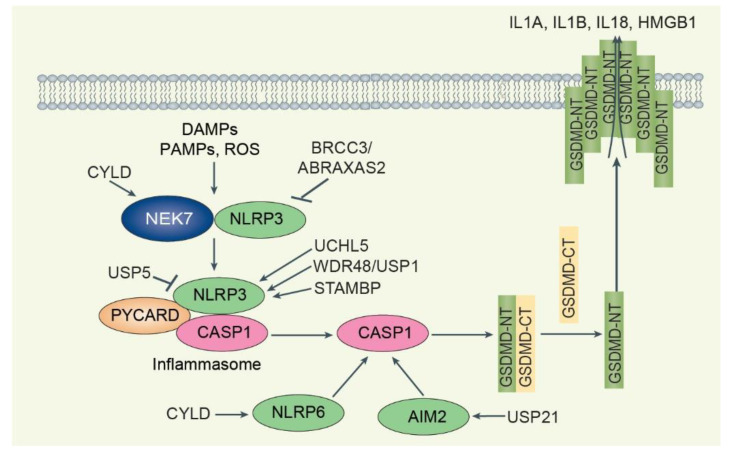
**Overview of DUBs-mediated regulation of pyroptosis**. DAMPs, PAMPs, and other stimuli promote inflammasome formation, such as NLRP3-PYCARD-CASP1. NEK7-NLRP3 binding prevents NLRP3 inflammasome formation. CYLD deubiquitinates NEK7 and promotes NEK7-NLRP3 association to inhibit NLRP3 inflammasome formation. While BRCC3/ABRAXAS2 deubiquitinates NLRP3 to promote NLRP3 inflammasome formation, the DUBs UCHL5, WDR48/USP1, and STAMBP promote NLRP3 inflammasome formation. USP5 promotes NLRP3 degradation and attenuates NLRP3 inflammasome activation. CYLD-NLRP6 and USP21-AIM2 regulate the NLRP6-PYCARD and AIM2 inflammasome, respectively. When inflammasome formation is activated, the inflammasome produces mature CASP1, which cleaves intact GSDMD. N-terminal GSDMD (GSDMD-NT) translocates to the inner leaflet of the plasma membrane, causing membrane rupture and release of DAMPs such as IL1A, IL1B, IL18, and HMGB1.

The canonical NLRP3 inflammasome is triggered by endogenous danger signals, such as pore-forming toxins, crystalline structures, extracellular ATP, and RNA, several pathogens (e.g., *Staphylococcus aureus*), and other factors (e.g., ultraviolet radiation) (Figure 5) [117]. Ubiquitination is crucial to control NLRP3 inflammasome activation. NLRP3 is maintained at low levels in inactivated cells because it is highly ubiquitinated by K63 and K48-linked polyubiquitin chains [122]. A series of E3 ligases, including membrane-associated ring-CH-type finger 7 (MARCHF7), SCF-FBXL2, BTRC, tripartite motif containing 24 (TRIM24), TRIM31, TRIM33, Ariadne homolog 2 (ARIH2), Cbl proto-oncogene B (CBLB), Ring finger protein 125 (RNF-125), Pellino-2 (PELI2), TRAF6, HUWE1, and Parkin, cooperate with DUBs to play a context-dependent role in regulating NLRP3 expression and activation [123]. Especially, NLRP3 undergoes sequential K63- and K48-linked polyubiquitination mediated by RNF125 and CBLB, respectively, which is essential for controlling its activation and, ultimately, endotoxemia and polymicrobial sepsis [124]. DUB BRCA1/BRCA2-containing complex subunit 3 (BRCC3) can directly bind to NLRP3 and cleave K63-linked polyubiquitin that is added by ubiquitin ligase RNF125 and subsequently facilitate NLRP3 inflammasome activation [124]. Despite evidence of normal priming, Abraxas 2, BRISC complex subunit (ABRAXAS2) knockout macrophages also phenotypically replicate BRCC3-deficient macrophages and exhibit impaired NLRP3-dependent processing of CASP1 [125]. ABRAXAS2-deficient cells display aberrant ubiquitination, inhibiting the interaction between NLRP3 and PYCARD, but not targeting NLRP3 for degradation. The association between ABRAXAS2 and BRCC3 and NLRP3 depends on the phosphorylation of NLRP3 serine 194 and the NLRP3 interactor NIMA-related kinase 7 (NEK7), which acts as a scaffold for bridging adjacent NLRP3 subunits and requires proposed priming [124,125]. Small molecule inhibitors of JAMM/MPN+ (thiolutin and holomycin) limit pyroptosis and inflammation induced by both wild-type and autoactivating NLRP3 mutants by inhibiting BRCC3 [126]. In contrast to BRCC3, DUB CYLD and its binding partner spermatogenesis associated 2 (SPATA2) inhibit the activation of the NLRP3 inflammasome [127]. CYLD deubiquitinates centrosome PLK4, leading to NEK7-PLK4 binding and NEK7 capture at the centrosome, which subsequently interferes with NLRP3-NEK7 binding and NLRP3 inflammasome assembly [127]. DUB TNFAIP3 also inhibits the activation of the NLRP3 inflammasome [128]. Unlike normal cells, TNFAIP3-deficient macrophages exhibit only spontaneous NLRP3 inflammasome activity against LPS. DUB UCHL5 promotes mycobacterial secretion of EST12 to activate the NLRP3 inflammasome. A tuberculosis protein called EST12 can induce macrophage pyroptosis, which then binds to the Tyr80-binding side of the activated endogenous host sensor protein receptor for activated C kinase 1 (RACK1) to form the EST12-RACK1 complex, which recruits the UCHL5 and causes K48-associated deubiquitination of NLRP3, followed by induction of GSDMD to induce macrophage pyroptosis and IL1B secretion [129]. Chemical inhibition of DUBs USP7 and USP47 increases the ubiquitination of NLRP3, prevents PYCARD oligomerization and speck formation, and blocks inflammasome formation [130]. The WD repeat domain 48 (WDR48, also known as UAF1), a cofactor that stimulates the DUB activity of USP1, USP12, and USP46, removes K48-linked ubiquitination of NLRP3, thereby preventing the proteasomal degradation of NLRP3 [131]. WDR48/USP1 inhibitor ML323 or deletion of the *Wdr48* gene ameliorates NLRP3-dependent inflammation in a folic acid-induced acute tubular necrosis model [132]. The DUB signal transducing adaptor molecule-binding protein (STAMBP), an endosome-resident DUB, negatively regulates NLRP3 inflammasome activation. Knockdown of STAMBP had no effect on NLRP3 protein abundance but increased NLRP3 K63 chain polyubiquitination, resulting in increased LPS-induced NLRP3 inflammasome activation. DUB USP5 attenuates NLRP3 inflammasome activation by selectively promoting K48-linked polyubiquitination of NLRP3 and mediating its degradation through the autophagy–lysosome pathway by recruiting the E3 ligase MARCHF7. USP5 overexpression by in vivo transfection reduces IL1B and polymorphonuclear infiltration in an alum-induced peritonitis model [133].

DUBs also regulate other types of inflammasomes. For example, DUB CYLD removes K63-linked polyubiquitin from NLRP6 to suppress inflammasome activation in mice infected with *Citrobacter rodentium* [134]. Deubiquitination by CYLD inhibits the formation of NLRP6-PYCARD inflammasome complex and, subsequently, the maturation and release of IL18. Ubiquitination and deubiquitination of AIM2 are key events that regulate AIM2 inflammasome activation via cytoplasmic double-stranded DNA. DUB ubiquitin-specific peptidase 21 (USP21) binds and deubiquitinates AIM2, which is required for AIM2 inflammasome assembly [135].

Taken together, the function of DUB in inflammasome and pyroptosis is dependent on external stimuli. Although the NLRP3 inflammasome is the most studied inflammasome in the past decade, the direct effect of ubiquitin on the pyroptotic mediator GSDMD remains uncertain.

## 7. Conclusions and Future Directions

Protein half-life that is too long or too short can affect cellular homeostasis and lead to human diseases, especially aging-associated diseases. Therefore, dissecting the dynamic process of protein ubiquitination and deubiquitination is crucial for understanding cell survival and cell death. Many key components of the cell death machinery can be fine-tuned by modification of ubiquitination and deubiquitination. The binding of ubiquitin to substrates occurs through a multi-step cascade consisting of E1, E2, and E3 enzymes. Like the vast family of E3 enzymes, DUBs are a large family that has context-dependent roles in regulating cell death by targeting different proteins. Briefly, DUBs-mediated ubiquitination of anti-injury proteins inhibits cell death. Conversely, DUBs-mediated deubiquitination of death mediators accelerates cell death. However, the specific target proteins and binding sites of key DUBs in mediating different cell death modalities induced by different stimuli remain incompletely understood. In theory, every protein should have a UPS mechanism to control its half-life. However, so far, not all key cell death mediators, such as GSDMD and MLKL, have well-defined DUBs to inhibit their protein degradation. Further understanding of these issues may be important for the development of highly selective drugs targeting DUBs. Regardless, understanding the tissue, cellular, and protein target specificity of DUBs in regulating cell death and other processes remains a challenge. We need further multidisciplinary collaboration on high-throughput screening of substrates, the development of activity-based probes to monitor target engagement, and, ultimately, DUB drug design.

## Data Availability

Not applicable.
